# Vitamin D status and the prevalence of deficiency in lactating women from eight provinces and municipalities in China

**DOI:** 10.1371/journal.pone.0174378

**Published:** 2017-03-23

**Authors:** Yao Zhao, Yingjie Yu, Hong Li, Zhirong Chang, Yongjin Li, Yifan Duan, Jie Wang, Shan Jiang, Zhenyu Yang, Shi-an Yin

**Affiliations:** 1 Beijing Center for Disease Control and Prevention, Beijing, China PR; 2 Beijing Research Center for Preventive Medicine, Beijing, China PR; 3 Beijing Dongcheng Center for Disease Control and Prevention, Beijing, China PR; 4 Beijing Shunyi Center for Disease Control and Prevention, Beijing, China PR; 5 National Institute for Nutrition and Health, Chinese Center for Disease Control and Prevention, Beijing, China PR; University of Alabama at Birmingham, UNITED STATES

## Abstract

**Background:**

Vitamin D deficiency has become prevalent worldwide in recent years. However, less evidence was available for lactating women.

**Objective:**

The purpose of the study was to understand vitamin D status and prevalence of deficiency in lactating women and associated risk factors for vitamin D deficiency from eight provinces and municipalities in China.

**Methods:**

Lactating women within 1–10 months postpartum were recruited in 2011–2013 from eight provinces and municipalities in China. Radioimmunoassay was used to measure serum 25-hydroxyvitamin D [25(OH)D] concentration. Standardized questionnaire was used to collect information on season, living site, ethnicity and socio-demographic characteristics.

**Results:**

Totally 2004 lactating women were recruited. The median (p25, p75) of 25(OH)D was 15.8 (10.5, 24.0) nmol/L. The prevalence of vitamin D deficiency was 85.3% as 25(OH)D <30nmol/L. Serum 25(OH)D levels of lactating women were significantly lower during October-January (14.0nmol/L) than during February-May (18.0nmol/L) (*P*<0.001), and were significantly higher in Dai ethnicity (22.5nmol/L) than in Hui ethnicity (Chinese Muslims) (9.0nmol/L) (*P*<0.001). For every 10,000 CNY annual income per capita increasing, serum 25(OH)D levels significantly increased 1.04 times (*P*<0.001). The odds of vitamin D deficiency in winter were 2.56 times higher than that in spring (OR 2.56, 95%CI: 1.91–3.43).

**Conclusions:**

Vitamin D deficiency of lactating women was highly prevalent in the eight provinces and municipalities in China. It is urgent to study the strategy and intervention ways for improving vitamin D status of lactating women, especially for certain population groups during low sunlight exposure season.

## Introduction

Vitamin D is a fat-soluble vitamin essential for metabolism and health outcomes such as bone mineralization and immune function. This vitamin could be provided by dietary intake as well as synthesized through the skin exposure to the ultraviolet from sunlight. Recently, the roles of vitamin D in health outcomes related to perinatal, lactating period and infants have been received considerable attention. Numerous studies have reported that low maternal vitamin D status is associated with multiple adverse obstetric outcomes [1–4].

Because breast milk is the source of both vitamin D and 25-hydroxyvitamin D [25(OH)D] for infants, maternal vitamin D status is an important factor in determining this vitamin status of infant and their risk of developing vitamin D deficiency such as infantile nutritional rickets [2, 5]. Breast milk is internationally recognized as the best sources of nutrition for optimal infant growth and development [3, 4]. Because maternal vitamin D status is the critical predicator of the vitamin D content in her breast milk, when a mother is vitamin D deficient, vitamin D content in her breast milk tends to be low. Therefore, given that vitamin D deficiency is a problem worldwide, breast milk generally does not contain sufficient vitamin D for breastfed infants [2, 5, 6]. Findings from epidemiological studies have indicated that low maternal vitamin D status and deficiency (serum 25(OH)D level <50 nmol/L) including pregnant and lactating women are increasingly recognized as a global public health problem[1]. Recent studies have showed that the prevalence of vitamin D deficiency in pregnant and lactating women was very high, for example, the prevalence of vitamin D deficiency (serum 25-hydroxy vitamin D_3_<75 nmol/L in the Pakistan study and serum 25-hydroxy vitamin D_3_<48 nmol/L in the China study) in some Asian women was as high as 98% [7–9]; about 72.6% of the healthy lactating Egyptian women were vitamin D deficiency [10]; the prevalence of vitamin D deficiency (serum 25(OH)D level <50 nmol/L) varied between 27%∼91% among pregnant women in USA and Canada and other countries[11–13].These results may partially explain why vitamin D deficiency and infantile rickets remain a major public health concern worldwide [2, 14].

Although maternal vitamin D status can affect not only their own nutrition and health status [1, 15, 16], but also vitamin D concentration in their milk [17–19], early mental and psychomotor development and growth of infants [17, 20, 21], fewer studies focused on vitamin D status of lactating women, compared with the studies in women of childbearing age [22, 23]. Sun exposure, skin color, dietary intake and BMI have been indicated to associate with vitamin D status. Exposure to sunlight is main source of vitamin D for adults. Sunscreen with sun protection factor of 8 or greater could reduce the cutaneous vitamin D synthesis significantly[24, 25]. Melanin pigment is associated with less ability to produce vitamin D. Lifestyle factors such as clothing and outdoor activities are also related to vitamin D status. In addition, altitude and seasonal variation could be risk factors for vitamin D deficiency. Vitamin D fortified food and vitamin D supplementation could improve vitamin D status significantly. Our purpose is to understand vitamin D status, prevalence of deficiency and associated risk factors related to vitamin D deficiency of lactating women from eight provinces or municipalities in China.

## Methods

### Study population

The study was approved by the ethic committee of the National Institute of Nutrition and Food Safety, Chinese Center for Disease Control and Prevention. Written informed consent was obtained from all lactating women.

To take geographic location and economic development into account (rural vs urban, coastal vs inland, and developing vs developed areas), Beijing and Shanghai municipalities, Guangdong, Heilongjiang, Yunnan, Gansu, Zhejiang and Shandong provinces were selected. Healthy lactating women at postpartum 30∼330 days were recruited to participate in this study.

#### Inclusion criteria

Lactating women were recruited with aged 20∼35 years old, any breastfeeding her baby, self-evaluated healthy, singleton pregnancy, no drinking and no smoking, her baby in healthy status, and signed informed consent.

#### Exclusion criteria

The following conditions were excluded, including mastitis, infectious diseases (tuberculosis, viral hepatitis and HIV infection, etc.), cardiovascular diseases, metabolic diseases such as diabetes, mental disorders, cancers and other malignant degenerative diseases, no ability to answer the question, currently participating in other studies related to nutrition or drug intervention, and refused to sign informed consent.

### Study design

This was a cross-sectional study. A standardized questionnaire was used to collect information from lactating women with face to face interview, which included socio-economic status (occupation, education, income, etc.), age, ethnicity, health and breastfeeding status, breastfeeding duration, survey date, times and latitude. The questionnaire also included the following information on pregnant and gestational information, personal life style, physical activity, 24-hour dietary recall, and food frequency questionnaire during the previous one month, the use of nutrient or dietary supplements, and medical history.

The survey was conducted in the fall and winter seasons (October, November, December and January) in Beijing municipality, Heilongjiang, Gansu and Shandong provinces, and in the spring season (February to May) in Shanghai municipality, Yunnan, Guangdong and Zhejiang provinces.

### Data collection procedures

Before or after the interview, body weight and height were measured in all lactating women to calculate body mass index (BMI), using standard anthropometric procedure. Height was measured to the nearest 0.1 cm, and body weight to the nearest to 0.1 kg. Before starting the field work, weight and height scales were calibrated. Overweight and obesity were classified using BMI cutoff (≥24 kg/m^2^ and ≥28 kg/m^2^, respectively) according to “the guidelines of overweight and obesity prevention and control for Chinese adult”. The study visits occurred at local health centers or a temporary assessment clinics setup within the local residential center (village or street committee).

### Blood sample collection and laboratory analysis

A fasting blood sample (5 mL) was drawn from an antecubital vein by local phlebotomist in the morning. The blood specimens were collected in serum separation tubes and immediately wrapped with aluminum foil and kept in the dark place for about 30–60 min, then centrifuged at 2500–3000 rpm for 10 min at room temperature. The serum fraction was then collected and placed in a refrigerator at-20∼-30°C at the field site. Once the fieldwork completed, all the serum samples were transported, in a frozen state, to Beijing by air and stored at -80°C until they were analyzed.

Commercial ^125^I-25(OH)D RIA kit (DiaSorin, Stillwater, Minnesota, USA) was used for analyzing serum 25(OH)D. vitamin D deficiency is defined as serum 25(OH)D<30 nmol/L. Vitamin D insufficiency is defined as serum 25(OH)D<50 nmol/L and ≥30 nmol/L. Vitamin D sufficiency is defined as serum 25(OH)D≥50 nmol/L[26].

### Statistical analysis

Double data entry was performed using Epi data 3.1. SAS 9.2 software was used for descriptive, bivariate and multivariate analyses. For those continuous variables which were not normally distributed, the data were analyzed after logarithmic transformation. Student t test or ANOVA was used for comparison of serum 25(OH)D concentrations among different levels such as latitude and education. Chi-square test was used to compare vitamin D deficiency among different levels such as seasons. General linear model was used to identify the factors associated with 25(OH)D level such as demographic characteristics, feeding time, survey date, time and latitude. Multiple logistic regression was used to analyze associated risk factors for vitamin D deficiency and stepwise regression method was used for model selection (SLE = 0.20, SLS = 0.05).

## Results

Participant characteristics were shown in Table 1. This survey recruited total of 2004 lactating women at postpartum of 1∼10 months who consented. The average age was 27.1 years and lactating duration was 5.3months. Overall, 72.5% of lactating women were Han ethnicity. The proportion of Bai, Hui (Chinese Muslim), Zang, Dai and other ethnicities were 5.8%, 6.4%, 6.4%, 6.2% and 2.7%, respectively.

**Table 1:** General characteristics of study participants.

Indicator	Beijing	Heilongjiang	Shanghai	Yunnan	Gansu	Guangdong	Zhejiang	Shandong
Sample size	249	472	228	350	368	191	16	130
Age (y)^1^	28.6±3.9	27.6±3.9	28.5±4.1	25.4±4.1	24.9±4.3	27.5±4.1	30.4±4.6	30.3±3.5
Height (cm)^1^	160.6±5.7	160.3±5.5	159.8±6.1	155.8±5.2	159.4±5.2	157.0±9.1	158.3±4.8	162.2±7.1
Weight (kg)^1^	61.0±9.5	60.3±10.3	58.7±9.2	52.4±8.0	52.8±7.0	54.8±8.4	56.5±5.0	63.4±9.6
BMI (kg/m^2^)^1^	23.6±3.4	23.4±3.8	23.1±4.4	21.6±3.1	20.8±2.4	22.1±3.2	22.4±1.9	24.2±4.9
Education(%)^2^								
≤9 years	12.8	44	22.8	90.6	90.2	42.4	31.3	10.8
10∼15 years	43.8	37.3	37.7	9.2	6.8	45.6	56.3	51.5
≥ 16 years	43.4	18.6	39.5	0.3	3.0	12.0	12.5	37.7
Professions (%)								
Headship^3^	7.6	7.2	7.5	0.9	0.5	2.1	18.8	3.9
Technician^4^	20.1	16.3	21.1	2.0	3.3	15.7	0.0	26.2
Staff^5^	14.5	4.9	13.6	0.0	3.0	12.0	6.3	13.1
Service^6^	22.9	12.1	12.3	4.9	3.3	7.3	18.8	20.0
Agriculture^7^	0.8	12.9	0.0	66.0	87.0	1.1	0.0	0.0
Homemaker	24.9	22.7	31.6	24.0	3.0	46.6	37.5	18.5
Others	9.2	24.0	14.0	2.3	0.0	15.2	18.8	18.5
Income(CNY)^8^	3.4±2.9	1.4±1.3	3.6±2.8	0.8±0.7	0.6±0.4	2.6±1.3	2.7±1.5	2.8±3.8

^1^The results were expressed as Mean±SD.

^2^Education, illiterate who don’t understand less than 1000 Chinese characters or an ability to read or write a note; ≤9years, elementary school and junior high school; 10∼15years, senior high school and college; ≥ 16 years university or above.

^3^Headship, person in charge of organs and enterprises and institutions.

^4^Technician, person mainly engaged in professional and technical working.

^5^Staff, person working in office such as government, organs and enterprises and institutions.

^6^Service, person working in commercial and service industries.

^7^ Agriculture, person working in agriculture, forestry and animal husbandry, fishery and water conservancy.

^8^Income was expressed as yearly family income of Chinese yuan (CNY) Renminbi (RMB) multiply 10 000.

The mean BMI was 22.5 kg/m^2^. The percentage of underweight, overweight and obesity were respectively 10.2%, 22.5% and 6.9%. Total of 44.9% of lactating women were from Southern part of China including Shanghai municipality, Yunnan, Guangdong and Zhejiang provinces with latitude ranging from North 21° to North 30°. The other lactating women were from North or northwest in China including Heilongjiang, Beijing, Gansu and Shandong provinces with latitude ranging from North 34°to North 48°.

Serum 25(OH)D concentration and percentage of vitamin D deficiency in lactating women were shown in Table 2. The median of serum 25(OH)D concentration was 15.8nmol/L and the values of 25th percentile and 75th percentile were 10.5nmol/L and 24nmol/L, respectively. Vitamin D deficiency and marginal deficiency were respectively 85.3% (25(OH)D<30 nmol/L) and 12.6% (25(OH)D 30∼50 nmol/L), and there were only 2.1% lactating women who had adequate serum 25(OH)D level (25(OH)D>50 nmol/L). Only 0.3% (6/2004) of lactating women had serum 25(OH)D level greater than 75 nmol/L. No significant association was observed between maternal 25(OH)D concentration and the course of lactation (r = -0.02, p = 0.45, n = 2004); when lactation stage was grouped as ≤3 mo, 3–6 mo and > 6 mo, the mean 25(OH)D concentration was not significantly different among the 3 groups (F-value = 0.50, p = 0.60) (data were not listed).

**Table 2:** Serum 25(OH)D concentration and deficient prevalence of lactating women^1^.

Site	Sample size, n	Days of postpartum	25(OH)D concentration nmol/L	Deficiency (%)^2^		
				VDIs	VDD	Total
Beijing	249	164±84	16.0±11.5	8.0	90.4	98.4
Heilongjiang	472	163±85	18.2±12.0	10.0	87.1	97.0
Yunnan	350	171±90	18.8±9.5	10.0	89.1	99.1
Gansu	368	166±86	14.0±12.2	6.5	92.4	98.9
Shanghai	228	154±84	21.0±10.8	16.2	82.5	98.7
Zhejiang	16	112±63	17.2±9.0	12.5	87.5	100.0
Guangdong	191	123±85	29.2±12.5	37.2	57.1	94.2
Shandong	130	147±94	17.8±12.2	12.3	84.6	96.9

^1^ The results were expressed as mean±SD.

^2^Vtamin D insufficiency(VDIs), serum 25(OH)D 30∼50 nmol/L; vitamin D deficiency (VDD), serum 25(OH)D <30 nmol/L).

Gansu province had highest prevalence of vitamin D deficiency (92.4%) and lowest mean serum 25(OH)D level (14±12.2nmol/L), and then Beijing was second. The lactating women from Guangzhou municipality had highest concentration of serum 25(OH)D (29.2±12.5nmol/L) and slight lower prevalence of vitamin D deficiency (57.1%) compared with the other places in this survey.

The comparison of serum 25(OH)D of lactating women between rural and urban in Beijing and Shanghai municipalities, Guangdong and Heilongjiang provinces was shown in Table 3. There was significant difference in serum 25(OH)D (*P*<0.001) and vitamin D deficiency (*P*<0.001) between urban and rural sites in Beijing, and the vitamin D status of lactating women was better in urban than in rural. Serum 25(OH)D level was marginally greater in urban areas than in rural areas in Shanghai. No significant difference on vitamin D status was found between urban and rural in Heilongjiang and Guangdong provinces.

**Table 3:** Comparison of serum 25(OH)D of lactating women between rural and urban areas^1^.

Site	Urban				Rural			
	n	Day of	Content	Deficiency	n	Day of	Content	Deficiency
		postpartum	nmol/L	%		postpartum	nmol/L	%
Beijing	133	161±78	17.4^3^ (12.2,25.2) ^2^	82%	120	170±92	9.6 (5.7, 14.6)	99%
Heilongjiang	233	159±81	14.8^3^ (10.4, 23.2)^2^	88%	240	165±88	15.5 (9.7,23.5)	87%
Shanghai	144	131±82	21.8^3^ (14.2, 28.5)^2^	78%	94	196±69	18.0 (12.0, 23.8)	88%
Guangdong	75	142±91	28.0^3^ (19.6, 35.0)^2^	56%	111	110±79	27.2 (21.2,37.5)	58%

^1^The numbers in parenthesis were 25 percentile and 75th percentile.

^2^ t-test on serum 25(OH)D content (urban vs rural), BJ p< 0.001; HLJ p = 0.86; SH p = 0.09; GZ p = 0.72.

^3^ Chisq on vitamin deficiency (urban vs rural), BJ p< 0.001, HLJ p = 0.67, SH p = 0.051, GZ p = 0.82.

The results based on bivariate analysis showed that significant association was found between serum 25(OH)D level and such factors including season, latitude, ethnicity, intake of foods rich in vitamin D and the use of supplements, education, occupation, and income (*P*<0.05). However, no significant association was seen between serum 25(OH)D level and obesity or between serum 25(OH)D level and outdoor activities.

The results of multivariate analysis on serum 25(OH)D concentration were shown in Table 4. Significant association was found between serum 25(OH)D and season, education level, ethnicity and income (*P*<0.05). The 25(OH)D level in spring was corresponding to 1.28 times higher than that in winter (18nmol/L vs 14nmol/L). For every 10 000 CNY increase of per capita income, serum 25(OH)D level increased by about 4%. Lactating women with senior high school or college education had significantly lower serum 25(OH)D level than those with junior high school education or less (14.2nmol/L vs 16.8nmol/L, *P* = 0.01; 14.8nmol/L vs 16.8nmol/L, *P* = 0.03). Serum 25(OH)D levels were significantly lower in Hui ethnicity than in the other ethnicities (*P*<0.001) and Dai’s lactating women had highest serum 25(OH)D levels (*P*<0.005).

**Table 4:** Factors associated with serum 25(OH)D concentration of lactating women in China^1^.

Factor	n (%)	25(OH)D^2^^,^ nmol/L	t(F)	P
Seasons			-4.13	<0.001
Winter	1380 (71.2%)	14 (2.5)		
Spring	557 (28.8%)	18 (2.8)		
Latitude			-0.95	0.34
North	1105 (55.1%)	15.2(2.8)		
South	899 (44.9)	16.5(2.5)		
Education^2^			7.63	<0.001
≤9years	1041(51.9)	16.2 (2.5)		
10∼15years	591(29.5)	14.5 (2.5)		
≥ 16 years	372 (18.6)	16.5 (2.5)		
Ethnicity			24.55	<0.001
Han	1439 (72.5)	16.2 (2.5)		
Zang	128 (6.4)	16.0 (2.8)		
Hui	127 (6.4)	9.0 (2.8)		
Dai	124 (6.2%)	22.5 (2.8)		
Bai	116 (5.8)	17.0 (2.8)		
Income^2^	1999	1.04 (1.0)	4.06	<0.001

^1^ Explanation as same as footnotes on education and income in Table 1.

^2^ Geometric mean (SE).

Associated risk factors for vitamin D deficiency were shown in Table 5. Factors associated with vitamin D deficiency were season (winter vs spring, OR = 2.56), ethnicity (Bai vs Dai, OR = 3.67; Zang vs Dai, OR = 1.44; Han vs Dai, OR = 2.23; others vs Dai, OR = 1.41), and income (OR = 0.92).

**Table 5:** Risk factors for vitamin D deficiency of lactating women in China.

Influencing factor	OR (95% CI)
Season (Winter vs Spring)	2.56 (1.91, 3.43)
Ethnicity	
Bai vs Dai	3.67 (1.52, 8.88)
Zang vs Dai	1.44 (0.73, 2.83)
Han vs Dai	2.23 (1.36, 3.64)
Hui vs Dai	7.25 (2.44, 21.56)
Others vs Dai	1.41 (0.60. 3.27)
Income^1^	0.92 (0.87. 0.98)

1 Explanation as the same as footnotes on income in Table 1.

## Discussion

Because the major circulating form of vitamin D in blood is 25(OH)D, the level of serum 25(OH)D is currently accepted as the best biochemical indicator to evaluate vitamin D status [27, 28]. However, the adequate level of circulating 25(OH)D required for optimal health is uncertain [3,8]. Some guidelines have been recommended in defining the cut-off point of serum 25(OH)D for evaluating vitamin D status [9–12]. Generally, most researchers agree that serum 25(OH)D levels of 30–50 nmol/L and <30 nmol/L are defined as vitamin D insufficiency and vitamin D deficiency, respectively [13–15]. The present study showed that a total of 85.3% and 12.6% of lactating women were vitamin D deficiency and marginal deficiency, respectively (Table 2), which account for 97.9%.

It has been reported that vitamin D deficiency is a major public health concern worldwide in all age groups particular in special physiological stage such as pregnancy and lactation[29], even in the populations living in those countries with low latitude, where it was generally assumed that UV radiation was adequate to prevent vitamin D deficiency, and in industrialized countries, where vitamin D fortification has been implemented for decades [14, 18, 20, 30]. However, few studies had been conducted in lactating women at national level. To our knowledge this is the first large-scale study on vitamin D status and analysis on associated risk factors for vitamin D deficiency of lactating women in China. The median of serum 25(OH)D was only 15.8 nmol/L which was much lower than that of the cutoff point (30nmol/L) to define vitamin D deficiency and also much lower than the means of lactating women reported previously in US [31], Thailand [30] and United Arab Emirates [32], and similar to those reported in lactating women living in Lahore [7]. Therefore, our results indicated that lactating women would be vulnerable groups suffered from vitamin D deficiency because such populations usually avoid direct exposure to sunlight and consume fewer foods naturally rich in vitamin D and national fortification of food with vitamin D has not been implemented in China.

The concentration of serum 25(OH)D was affected by many factors such as season or the extent of exposure to UV-B radiation, use of vitamin D supplements, intake of diets rich in vitamin D and use of estrogen contraceptives as well as the difference between urban and rural areas (Table 3 and Table 4) [30, 33–36]. There was higher serum vitamin D level of lactating women in urban area than in rural area in Beijing, which might be due to the difference of multivitamin supplement uses as well as the intake of food rich in vitamin D [33]. Season is an important factor in determining vitamin D status of lactating women which could be related to sunlight exposure. Several studies have found that 25(OH)D levels and vitamin D deficiency were significantly associated with the season [33, 34, 36]. Compared with the summer and fall, 25(OH)D levels were generally lower and prevalence of vitamin D deficiency was higher in winter and spring [37]. It has been reported that the efficiency of vitamin D conversion in skin exposure to sunlight is near to zero in the northern parts during winter, even though at 11 am to 3 pm the maximal conversion rate of 7-dehydrocholesterolto vitamin D in skin is only about 1% [38]. Our study found that there was relatively higher serum 25(OH)D concentration in spring, which indicated that a higher conversion rate would have in this season. The conversion efficiency of vitamin D is generally higher at lower latitudes than at higher latitude regions, present survey also found that mean 25(OH)D levels of lactating women in southern part such Guangzhou city and Shanghai municipality were significantly higher than that in northwest region such Gansu province. Demarcation between Southern and Northern parts of China is based on the boundary Qinling Mountain and Huaihe River, so that Shanghai municipality, Guangzhou city of Guangdong province and Zhou-shan city of Zhejiang province are designated as the South. Because these areas locate in south away from 30 degrees of north latitude, the conversion efficacy of vitamin D in winter is also low which could narrow the gap between north China and south China. Therefore, higher vitamin D intake or supplements in lactating women would be needed during the winter at northern latitudes to avoid vitamin D deficiency [19, 20, 23].

We also observed that education level and household income were associated with vitamin D status in lactating women (Table 4), lactating women with senior high school or college education had significantly lower serum 25(OH)D level than that of the women with junior high school education or less that was different from the results in Netherlands [39] and Spain [17].The opposite result could be explained by the proportion of higher-educated lactating women who usually had work indoors and had less outdoor activities [30]. It has been reported that the mean level of 25(OH)D was associated with household income [30]. Our study showed that serum 25(OH)D level could be increased by about 4% following 10 000 CNY increase of per capita income (Table 4) which was contrary to the study in pregnant women in Thailand [30]. This might be related to food intake rich in vitamin D.

The present study found that serum 25(OH)D level of lactating women in Dai ethnicity was significantly higher than those of the other nations, because lactating women of Dai’s nation are living in Xishuangbanna minority autonomous region where is located at the latitude between 21∼22 degrees of the North. Even though serum 25(OH)D concentration in Dai’s lactating women is the highest among all nations, their average level is only 22.5 nmol/L which is much lower than the cutoff value for vitamin D deficiency (30nmol/L) too. These results further indicated that vitamin D deficiency in lactating women is much more widespread in spring in China, including even as Dai’s residents living in the tropics are no exception [30]. Serum 25(OH)D levels of lactating women in Hui ethnicity (Chinese Muslin) were the lowest among all nationalities which might be related to their dressing habits or customs [40]. Therefore, vitamin D intervention should be considered as priority. We can target to such populations who had higher education with high school, college and degree to increase their knowledge how to prevent vitamin D deficiency.

Based on bivariate analysis, our results indicated that the intake of food rich in vitamin D such as seafood and use of nutrient supplements were associated with the higher serum 25(OH)D level which agreed with previous studies in lactating women[5, 20]. However, by multivariate analysis, such factors showed no longer significant determinants of vitamin D status, which indicated that such factors would have little contribution to vitamin D status compared with skin exposure to the sun light and ethnicity which were the main determinants [41]. It has been reported that obesity in adults would increase the risk suffered from vitamin D deficiency [42], but our study found that serum 25(OH)D levels in lactating women were not associated with their BMI. A study from United Kingdom also showed that BMI value of adults aged 20∼40 year-old was not associated with serum25(OH)D levels, but significant association between both was found for those persons aged 64 years or more[43]. It needs to mention that serum 25(OH)D concentrations from present study were significantly lower than that of UK’s study, which also suggested that the relationship between BMI and serum 25(OH)D levels might be dependent upon the vitamin D status.

Recently, it has been shown that maternal supplementation beginning in gestation with 50 μg vitamin D_3_/d could protect 98% of breastfed infants without vitamin D supplementation against 25(OH)D deficiency (<30 nmol/L) to at least 8 weeks in Canada [20]. However, current Chinese recommended dietary intake (2013) as well as most of the other countries of vitamin D for lactating women is only 10 μg/d [23]. Further studies are needed to determine the optimal doses and duration of vitamin D supplementation during perinatal and lactation period [19], to understand the factors that are likely to impact vitamin D status, and to evaluate the effect of air pollution containing ozone on the reduction of cutaneous photosynthesis of previtamin D_3_[44].

Our study had some limitations. In this cross-sectional design, lactating women were voluntarily recruited so that the results could not be true representative of vitamin D status in lactating women at national level. Due to the special nature of lactating women, recruitment would be generally considered to be more feasible and acceptable. Through our relatively larger sample size, the present results, at a certain extent, could reflect the vitamin D status for surveying population. In addition, our study did not collect information on clothing style and sunlight exposure so that we could not give more accurate description on the differences between different ethnic groups and habits in wearing clothes. Exclusively breastfeeding were not distinguished from partially breastfeeding in the analysis, which may underestimate the vitamin D status. Our further study could add other novel metabolites (e.g. 20(OH)D) of vitamin D in the study [45–47], which may explain the relationship of vitamin D deficiency in lactating women to immune function and health status or/and infection rates through the solely activated pathways and metabolic mechanism of vitamin D.

In summary, vitamin D deficiency in lactating women was highly prevalent in winter and spring regardless of them living in south or the north in China. Vitamin D status in lactating women was mainly associated with season, ethnicity and income. It is urgent to study the strategy and implement interventions on vitamin D supplementation for improving vitamin D status of lactating women.

## Supporting information

S1 File Plosvdd.sas7bdat. (XLS)
